# Comparative transcriptomic analysis of the nodulation-competent zone and inference of transcription regulatory network in silicon applied *Glycine max* [L.]-Merr. Roots

**DOI:** 10.1007/s00299-024-03250-7

**Published:** 2024-06-12

**Authors:** Sheikh Mansoor, Pooja Tripathi, Amit Ghimire, Saira Hamid, Diaa Abd El-moniem, Yong Suk Chung, Yoonha Kim

**Affiliations:** 1 Department of Plant Resources and Environment, Jeju National University, Jeju, 63243 Republic of Korea; 2 Department of Applied Biosciences, Kyungpook National University, Daegu, 41566 Republic of Korea; 3 Department of Horticulture and Crop Science, The Ohio State University, Columbus, OH USA; 4 Department of Integrative Biology, Kyungpook National University, Daegu, 41566 Republic of Korea; 5 Watson Crick Centre for Molecular Medicine, Islamia University of Science and Technology, Awantipora, Pulwama, J&K India; 6 Department of Plant Production (Genetic Branch), Faculty of Environmental Agricultural Sciences, Arish University, El-Arish, 45511 Egypt

**Keywords:** Soybean, Silicon (Si) application, Transcriptomic analysis, Nodulation, Root development, Transcription factors (TFs)

## Abstract

**Key message:**

The study unveils Si's regulatory influence by regulating DEGs, TFs, and TRs. Further bHLH subfamily and auxin transporter pathway elucidates the mechanisms enhancing root development and nodulation.

**Abstract:**

Soybean is a globally important crop serving as a primary source of vegetable protein for millions of individuals. The roots of these plants harbour essential nitrogen fixing structures called nodules. This study investigates the multifaceted impact of silicon (Si) application on soybean, with a focus on root development, and nodulation employing comprehensive transcriptomic analyses and gene regulatory network. RNA sequence analysis was utilised to examine the change in gene expression and identify the noteworthy differentially expressed genes (DEGs) linked to the enhancement of soybean root nodulation and root development. A set of 316 genes involved in diverse biological and molecular pathways are identified, with emphasis on transcription factors (TFs) and transcriptional regulators (TRs). The study uncovers TF and TR genes, categorized into 68 distinct families, highlighting the intricate regulatory landscape influenced by Si in soybeans. Upregulated most important bHLH subfamily and the involvement of the auxin transporter pathway underscore the molecular mechanisms contributing to enhanced root development and nodulation. The study bridges insights from other research, reinforcing Si’s impact on stress-response pathways and phenylpropanoid biosynthesis crucial for nodulation. The study reveals significant alterations in gene expression patterns associated with cellular component functions, root development, and nodulation in response to Si.

**Graphical abstract:**

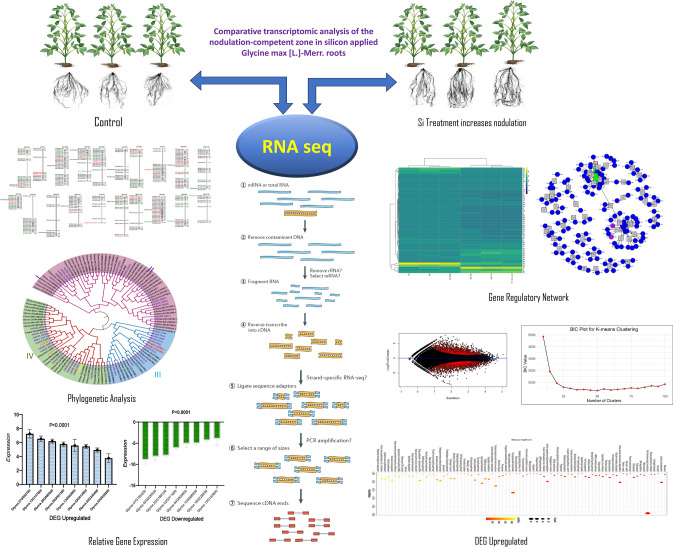

**Supplementary Information:**

The online version contains supplementary material available at 10.1007/s00299-024-03250-7.

## Introduction

Cultivated soybean (*Glycine max* (L.) Merr.), listed as an important legume and oilseed crop, has been widely used for human consumption and animal feed (Hartman et al. 2011). Among different plant parts, regarding the absorption of nutrients and water, roots play a pivotal role in soybean plants (Kim et al. 2021). The roots of leguminous plants like soybeans possess specialized structures known as nodules which form a symbiotic nitrogen-fixing relationship with the bacteria; Rhizobium. The interaction between the legume roots and the nitrogen-fixing rhizobia leads to the development of these specialized symbiotic structures (Nishida and Suzaki 2018; Kim et al 2023). The primary function of these nodules is to transform atmospheric nitrogen into ammonia or similar nitrogen-containing compounds within the rhizosphere (Tripathi et al. 2021). The nodules are extremely advantageous for plants since they offer a readily available supply of crucial chemicals that support and enhance plant growth (Suzaki et al. 2015). This symbiotic relationship between the plant and bacteria optimizes the nitrogen demand and nodule activity through metabolic regulation, oxygen control, production of reactive oxygen species (ROS) and reactive nitrogen species (RNS), and molecular adjustments (Schwember et al. 2019; Mansoor et al. 2022a, b). Consequently, the presence of root nodules plays a crucial role in supporting the well-being of plants. On the other hand, plants consistently confront unfavourable environmental conditions that pose limitations on their growth and productivity. As a result, plants have developed intricate adaptive mechanisms to respond to adverse environmental factors. Multiple studies have indicated that these adaptive mechanisms function through the control of gene expression, involving the activation or suppression of regulatory genes (Chen et al. 2002; Kalde et al. 2003; Mansoor et al. 2023). The nodule numbers in legumes are primarily regulated through a process known as autoregulation of nodulation (AON), and this process is further regulated by CLAVATA1-like receptor kinas (Reid et al. 2011). For the initiation of nodule development in legumes, nodulation (Nod) factors are the main signalling molecules, and most of these Nod factors have a core structure of three, four or five β-1,4-linked N-acetylglucosaminyl (Broughton et al. 2000; D’haeze and Holsters, 2002).

Silicon (Si) is an essential mineral element for the plants as its supplementation modulates the gene expression and signalling pathways, thereby enhancing root and nodule development, fortifying plant defence mechanisms, and augmenting yield output (Deshmukh and Bélanger 2016; Tayade et al. 2022). In natural conditions, Si typically exists as silica (SiO_2_) or various aluminosilicate forms (Brahma et al. 2020). Plants absorb Si from the soil primarily in the form of soluble silicic acid (H_4_SiO_4_). Specialized transporters facilitate Si uptake from the root zone. Once inside the plant, Si is transported and deposited in various plant tissues, primarily in the form of silica (SiO_2_). It accumulates in the epidermal cells, cell walls, and other plant structures (Sharma et al. 2023). Si deposition in plant cell walls contributes to their rigidity and resistance to biotic and abiotic stresses. Moreover, Si treatments on soybean plants have been shown to induce greater root length, diameter, and biomass (Guntzer et al. 2012). Si can promote root growth and branching, aiding in nutrient and water uptake and is particularly beneficial under conditions of stress (Srivastava et al. 2022). Si applications can influence the development of root nodules in leguminous plants, potentially enhancing nitrogen fixation and nutrient acquisition (Liang et al. 2015; Tripathi et al. 2022). At the cellular level, Si enhances the extensibility of cell walls in root growth zones, a factor that may contribute to increased root elongation and improved mechanical properties (Hattori et al. 2003). The molecular implications of Si application are profound, with evidence of significant modulation of gene expression in roots, as observed in *Brassica napus*, where Si supply leads to the alteration of the root transcriptome (Haddad et al. 2019). Additionally, Si improves root morphological characteristics by strengthening the antioxidative defence system, thereby reducing oxidative stress in root tissues (Ur Rahman et al. 2021). Apart from alleviating the biotic and abiotic stress Si enhances the interaction between legumes and rhizobia, leading to greater root nodulation, higher bacteroid counts, and increased nitrogen fixation in various legumes (Putra et al. 2020). Si has been implicated in the induction of nodule formation, which is crucial for biological nitrogen fixation and plant growth, particularly in legumes (Chung et al. 2020). A study conducted by (Nelwamondo and Dakora (1999) on *Vigna unguiculata,* where the application of Si resulted in a significant increase in nodule number, nodule dry matter, and resultantly higher nitrogen fixation. The same results were reported in another study where a lower concentration (50–100 µg) of Si supplementation resulted in a significant increase in nodule growth, among other traits (Mali and Aery, 2008). Building on the previous research, Putra et al. (2020) expanded that Si application not only increases nodule numbers but also its internal structure, which potentially affects its permeability and diffusion resistance (Putra et al. 2020; Sheehy et al. 1985). Although considerable literature is present on the positive role of Si application on root nodulation, there seems a variation in response to applied Si concentration. For example, a study reported that a moderate concentration Si application significantly increases the number of bacteroid and symbiosis, however, the higher concentration resulted in an increase in nodule cell wall thickness (Nelwamondo et al. 2001), which is a characteristic of Si accumulation and integration (Kumar et al. 2017), further confirmed by Garg and Singh (2018). Interestingly, Kurdali et al. (2019) reported that Si’s influence on root nodulation and nitrogen fixation in *Sesbania* legume was markedly enhanced under conditions of salinity and/or water stress, though these effects were lessened in the absence of such stressors. Furthermore, Garg and Singh (2018) demonstrated that Si facilitated nodule function through the upregulation of leghemoglobin production in pigeon pea genotypes, a hemoprotein critical for modulating oxygen levels within nodules, thus safeguarding the oxygen-sensitive enzyme nitrogenase and ensuring adequate oxygen for bacteroid respiration (Garg and Singh 2018; Ott et al. 2005).

Despite numerous reports on Si-induced modifications in root architecture, a detailed molecular understanding of how Si affects root nodulation remains elusive, particularly concerning the specific genes involved, their expression profiles, and the transcription factors (TFs) that regulate nodulation processes in response to Si. This includes a dearth of information about the involvement of distinct genes, their expression patterns, or the TFs responsible for regulating the process of nodulation in response to Si application. Therefore, in this study, RNA-Seq technology was utilized to investigate the changing patterns of gene expression and pinpoint the significant differentially expressed genes (DEGs) associated with soybean root nodulation induced by Si application. By conducting a comparative transcriptome analysis of soybean root nodulation, our aim was to unveil the evolving patterns of gene expression and detect the key DEGs/TFs, as well as metabolic pathways that play a role in the root nodulation formation in soybean upon Si application.

## Materials and methods

### Experimental design

The experiment was carried out in the year 2020 at the greenhouse located at Kyungpook National University, South Korea. Soybean variety Pungsannamul was used in the experiment. The Pungsannamul variety was selected as it showed higher efficiency of Si uptake in compared to other cultivars (Park et al. 2019). The soybean seeds were first sterilized using 70% ethanol from Sigma-Aldrich (USA) and then washed with double-distilled water before sowing. The experimental design followed a completely randomized setup with a fixed position arrangement. For each cultivar, two seeds were planted in each pot, later on thinned to one plant per pot, and this setup was replicated three times (*n* = 5). Once the plants reached the vegetative growth stage 1 (V1) they were randomly divided into two groups: control and treatment group. The treatment group received pure solution of Si, i.e. sodium metasilicate (Na_2_SiO_3_; Sigma-Aldrich, USA) and distilled water making a total volume of 100 ml through soil drenching. Conversely, the control group was irrigated with 100 ml of plain water only. The treatment was applied continuously for 10 days. For the investigation of root nodulation, samples were collected at the end of the experiment.

### cDNA library construction and transcriptome sequencing

The workflow for RNA-Seq Data Generation is depicted in Supplementary File S1. The total RNA [2 milli gram (mg)] was sent to PHYZEN (Gyeonggi-do, Republic of Korea) for sequencing, assembly and clustering analysis. The total RNA was quantified and qualified using an Agilent 2100 Bioanalyzer (Agilent Technologies, Palo Alto, CA, USA) and a NanoDrop system\ (Thermo Fisher Scientific Inc., Waltham, MA, USA). 1 mg total RNA with RIN value above 7 was used for cDNA library preparation. First, double-strand cDNA was synthesized, and then treated to repair both ends and add a dA-tailing, followed by a T-A ligation to add adaptors to both ends. Size selection of adaptor-ligated DNA was then performed using AxyPrep Mag PCR Clean-up (Axygen, Union City, CA, USA), and fragments of ~ 200–400 bp were recovered. The PCR products were cleaned up using AxyPrepMag PCR Clean-up (Axygen, Union City, CA, USA), validated using an Agilent 2100 Bioanalyzer (Agilent Technologies, Palo Alto, CA, USA), and quantified by Qubit 2.0 Fluorometer (Invitrogen, Carlsbad, CA, USA). Then libraries with different indices were multiplexed and loaded on an Illumina HiSeq instrument according to the manufacturer’s instructions (Illumina, San Diego, CA, USA). Sequencing was carried out using a 2 × 150 bp paired-end (PE) configuration.

### Samples and biological replicates quality check

'PtR' (https://github.com/trinityrnaseq/trinityrnaseq/wiki/QC-Samples-and-Biological-Replicates) a script for investigating the relationship between replicates and samples by performing transcript quantification on each biological replicate. In case there are errors between replicates and samples, such as mis-labeling, strong outliers, batch effects, etc., this is a way to correlate them before moving on to the next analysis (differential expression, etc.). The sample 1_NO, 2_NO and 12_NO_mx are control and 3, 4, 34_mx are treatment.

### RNA-seq data analysis

To increase the accuracy of the analysis, the raw data obtained after sequencing was pre-processed. Bioinformatics tools such as FastQC and Trimmomatic ( http://usadellab.org/cms/index.php?page=trimmomatic) were used to check the quality of raw data and eliminate low quality reads and adapter sequences. For organisms with known genetic information reference genome,

(https://phytozome.jgi.doe.gov/pz/portal.html#!info?alias=Org_Gmax), RNA-Seq reads were mapped to the sequence and used for the next step of analysis. The read alignment (mapping) of this analysis was performed using HISAT2 (http://daehwankimlab.github.io/hisat2/). Mapping data was used to perform differentially expressions. Compare gene expression between samples by counting the mapped reads using HTSeq-count and calculating the DESeq Normalization value based on the count value. Based on the DESeq Normalization value, the function was predicted for the selected DEG and GO analysis was performed for genetic interpretation and utilization. To increase the accuracy of the analysis results, the ‘Trimmomatic’ program was used to perform quality trimming (including removal of adapters) of raw data. The ‘Trimmomatic’ option applies to minimum quality of base (Bolger et al. 2014), sliding window (Lun and Smyth 2014), average quality (Anders et al. 2013), and minimum read size (Khang and Lau 2015). The degree of increment or decrease of the data was variable depending on the raw data quality obtained. The changes in the number of reads, percentage of high-quality base (Q 30%), and throughput of the raw data and trimmed data generated after the process. In addition, the analyzed results were visualized using various bioinformatics tools. The R statistical package, which is used for the visualization of RNA-Seq analysis and quality control measurements, supports statistical analysis, rapid data processing, and graphing.

### Phylogenetic tree construction and sequence analysis

Multiple sequence alignment of domain sequences of Glyma and HLH family proteins from soybean and 70 protein sequences from *Arabidopsis* was performed using the Clustal X 1.83 program with default parameters, and a phylogenetic tree was generated and viewed using MEGA Version 5.0. Exon and intron organizations of soybean HLH genes were determined by comparing predicted CDS with their corresponding genomic sequences.

### Gene ontology (GO) analysis and KEGG enrichment analysis

Gene Ontology (GO) analysis and Kyoto encyclopaedia of genes and genomes (KEGG) analysis were performed on reference genes. GO distinguishes various phenomena that can occur in living organisms into three categories (biological process, molecular function, and cellular component), and based on this, it is possible to infer the functions of each selected gene. The KEGG analysis was performed in two ways. First, we imported the results of performing Blast based on the nr database from the Blast2go program, and then found the matching KEGG pathways (KEGG_SEQ_by_Blast2GO, KEGG_Pathway_by_Blast2GO). Another method was to analyze the protein sequence of the reference gene on the KAAS web-tool (https://www.genome.jp/kegg/kaas/) to find a matching KEGG pathway (KEGG_by_KAAS). In this analysis, the modified Fisher’s exact test value was calculated using the R program, and the *P*-value value was calculated for each pathway.

### iTAK program for transcription factor (TF) sequence search

The Feizen Information Analysis was used find and classify all types of TF genes in the entire unigene sequence, automated transcription factor searches and classification by (http://itak.feilab.net/cgi-bin/itak/index.cgi) which provide better results than manual search using BLAST analysis (Zheng et al. 2016). The soybean TFs were discovered using the iTAK program in this analysis.

### Quantitative real time-PCR analysis and statistical tests

The methods for quantitative real time-polymerase chain reaction (qRT-PCR) analysis and statistical tests were performed as described previously (Zhao et al. 2018; Masoodi, et al. 2022). The relative gene expression was evaluated as previously described (Livak and Schmittgen 2001).

### Inference of gene regulatory networks

#### Expression clustering

Gene expression levels for each gene were normalized using DESeq2 and summarized as fragments per kilo-base pair per million reads values (FPKM values). The gene expression levels (FPKM values) were averaged across replicates and only DEGs were used in the clustering analysis. K-means clustering (Sherlock 2000) was performed using R packages, and the number of clusters (K) was determined using the minimum Bayesian Information Criteria (BIC) method (Ramsey et al. 2008).

#### Network inference methods

To infer regulatory networks, we adopted the methods of module networks (Segal et al. 2003). First, genes were grouped into modules using the K-means clustering method. Second, differentially expressed TFs were used as putative regulators for network inference. In our data, we found 82 clusters (gene modules) and 954 TFs that were differentially expressed in at least one comparison. The mean expression profile for each of the 82 modules was computed and the expression levels of 954 TFs were included to construct an expression matrix with 1036 rows (genes) and 6 columns (six experimental lines). Four network inference algorithms: ARACNE (Margolin et al. 2006), LARS (Haury et al. 2012) partial correlation (Schäfer and Strimmer 2005) and CLR (Faith et al. 2007) were applied to this expression matrix to infer putative regulatory interactions between each TFs and gene modules. The methods were chosen because it represents a diverse set of computational methods for gene network inference. The analysis was done using latest version of the R software.

### Data analysis

#### Experimental design and statistical analysis

The presented data represent mean values obtained from three independent experiments. Statistical analyses were conducted using GraphPad Prism 8. Duncan’s multiple test and analysis of variance (ANOVA) were performed using SAS 9.0 to assess the significance of differences.

#### RNA-seq data analysis

For the RNA-seq data, ClusterProfiler (version 3.4.4) software was employed to conduct enrichment analysis of the KEGG pathway for DEGs. Statistical significance was determined at the *p* < *0.05* level.

## Results

### Phenotype identification and transcriptome sequencing

**Fig. 1:**
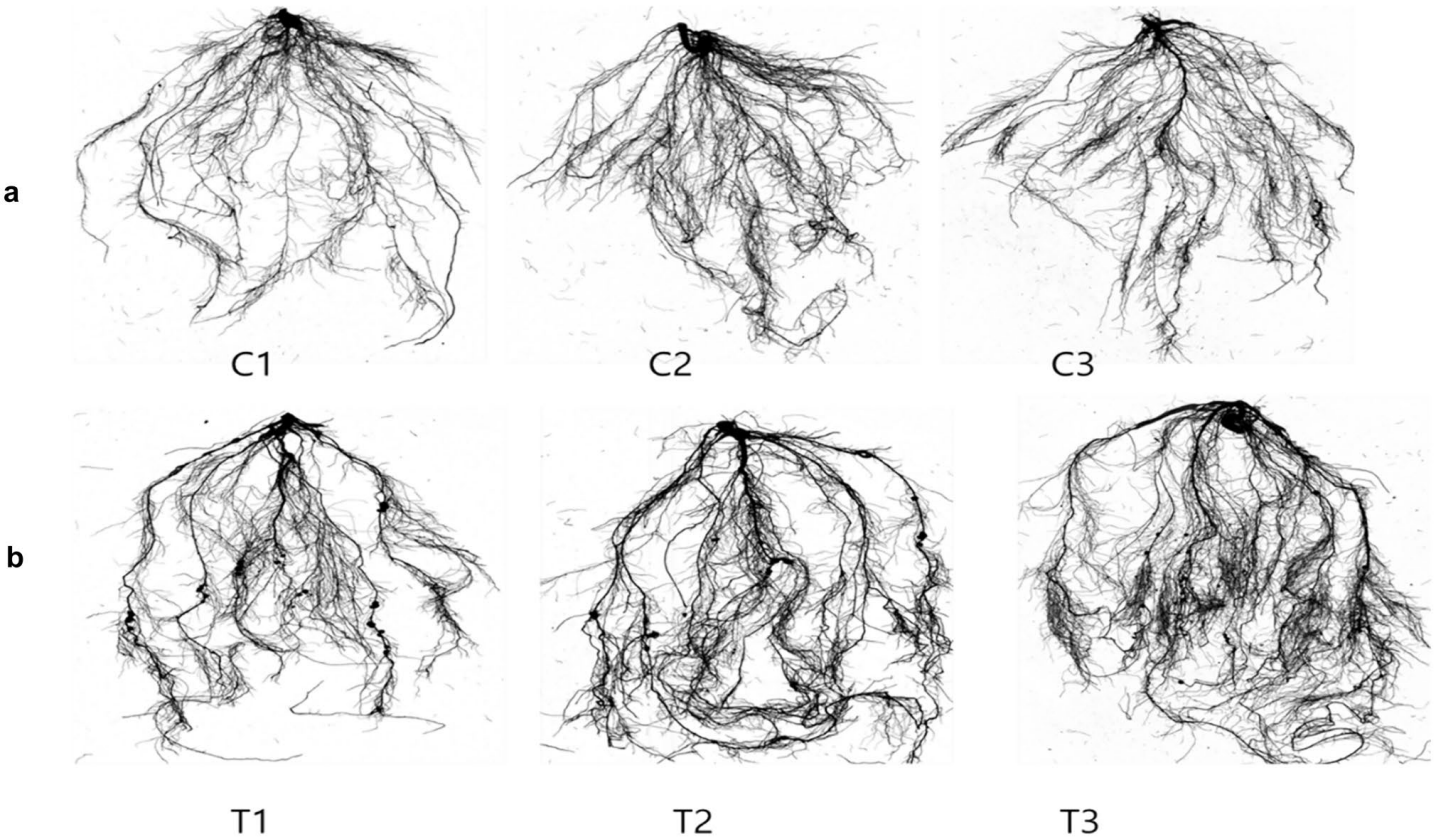
**A** Root nodulation and root hair density observed in control groups (C1–C3), and **B** treatments with Si application (T1–T3)

To investigate alterations in both root nodulation and gene expression following the application of Si to soybean roots, RNA sequencing data was generated and subsequently analyzed through transcriptome sequencing at the end of the experiment. The results revealed that the application of Si not only led to a significant augmentation in nodulation but also resulted in an increased root density in soybean plants. These phenotypic findings underscore the impact of si supplementation on both the molecular and morphological aspects of soybean root development, providing valuable insights into the underlying mechanisms and potential applications in optimizing soybean growth **(**Fig. 1).

Following the sequencing process, an extensive volume of data was generated, with the sequencing data summarized in Table 1. A total of 9.31 million and 10.03 million clean reads were obtained from the control and treated samples, respectively. Subsequently, these reads underwent trimming and alignment against the reference genome. Notably, a high degree of alignment to the reference genome was observed, as evidenced by statistical results indicating that 26,926,144, 32,117,278, and 33,692,762 reads were mapped for control samples, and 34,246,320 and 37,522,180 reads were mapped for treated samples, with an average matching rate of 91.51%. To further assess the sequencing depth and evenness, an analysis of transcriptome saturation and evenness was conducted.

**Table 1:** Summary of soybean root transcriptome data

Sample	Reads	Read bases	GC (%)	AT (%)	Q20 (%)	Q30 (%)
1_NO	26,926,144	4,065,847,744	46.41	53.59	97.41	93.51
2_NO	32,117,278	4,849,708,978	46.09	53.91	97.46	93.63
3	29,153,526	4,402,182,426	45.88	54.12	97.66	94.05
4	33,692,762	5,087,607,062	45.92	54.08	97.59	94.00
12_NO_mx	34,246,320	5,171,194,320	46.39	53.61	97.37	93.86
34_mx	37,522,180	5,665,849,180	45.30	54.70	97.63	94.43

**Fig. 2:**
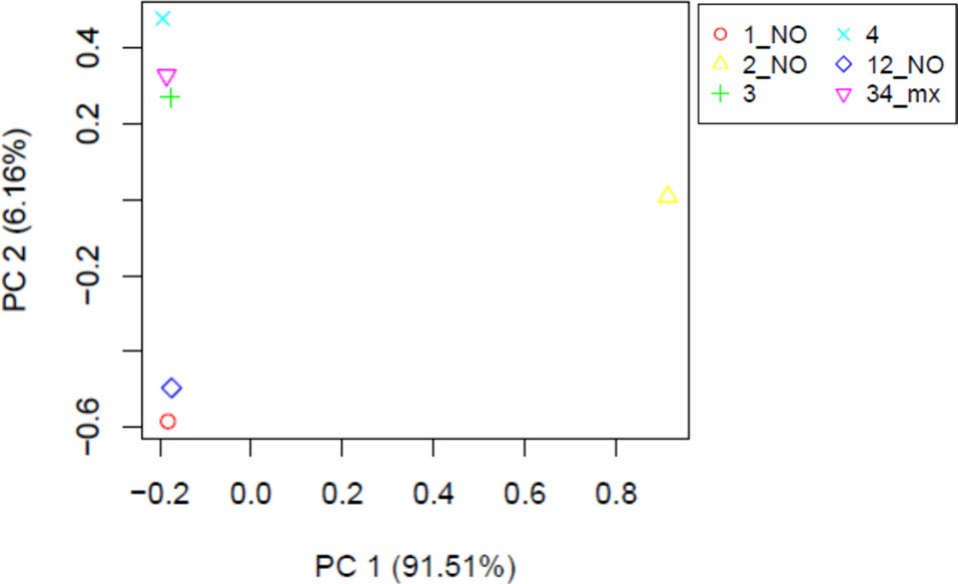
The relationships between biological replicates and samples by conducting PCoA based on transcript quantification for each replicate

The assessment of evenness in expression was conducted across entire transcripts, spanning from the 5′ to the 3′ end. The findings indicate a consistently high quality of sequencing throughout the study, encompassing a substantial portion of expressed genes. Furthermore, correlation analysis among various samples reveals an average correlation exceeding 91.5% (Fig. 2). As illustrated in the accompanying figure, proximity correlates with higher sample similarity, and replicates’ resemblance can be discerned through variance patterns. The input values for the PtR program consist of the expression values for each sample across the entire gene set. This statistical approach allowed for the exploration of correlations and patterns within the data, shedding light on the interplay between replicates and individual samples. The quantification of transcripts in each biological replicate served as the basis for assessing the correlation through principle coordinates analysis (PCoA), providing valuable insights into the underlying dynamics and relationships present in the dataset.

### RNA-Seq read mapping

**Fig. 3:**
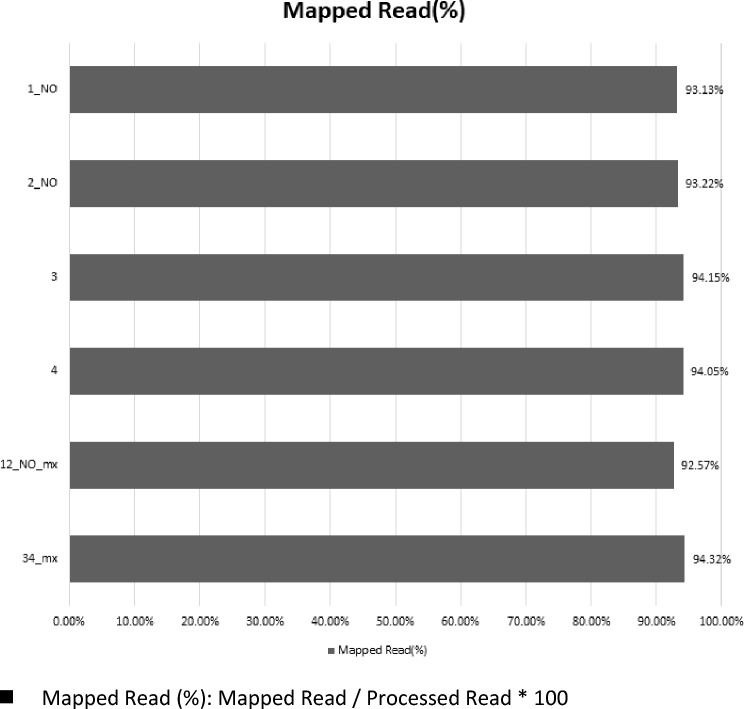
Mapped Reads Proportion: Graphical representation illustrating the efficiency of RNA-Seq read alignment

The genomic composition encompasses 20 chromosomes and 1170 scaffolds. Within this genomic landscape, a total of 56,044 transcripts, coding sequences (CDS), and protein sequences were identified, with exclusive utilization of primary transcript data. The analysis employed RNA-Seq reads, aligning them to the genome sequence, and specifically focusing on those reads mapped to the transcript sequence region. This subset of mapped RNA-Seq reads was then quantified and utilized for subsequent analyses. The genome comprises 1190 sequences with a cumulative base pair length of 978,495,272. Among these, there are 56,044 transcript sequences encompassing 92,028,847 base pairs, and 56,044 protein sequences with a combined base pair length of 21,820,865 (Supplementary File S1). Utilizing HISAT2, the RNA-Seq reads underwent alignment and mapping to the reference sequence. The accompanying Fig. 3 visually depicts the proportion of mapped reads for each sample, serving as a graphical representation of the efficiency of RNA-Seq read alignment concerning both the reference genome and transcript sequences. Notably, the mapped reads exhibit a substantial alignment, ranging from 92 to 94% as can be seen in Fig. 3.

### Identification and distribution of the Glyma gene family in soybean

**Fig. 4:**
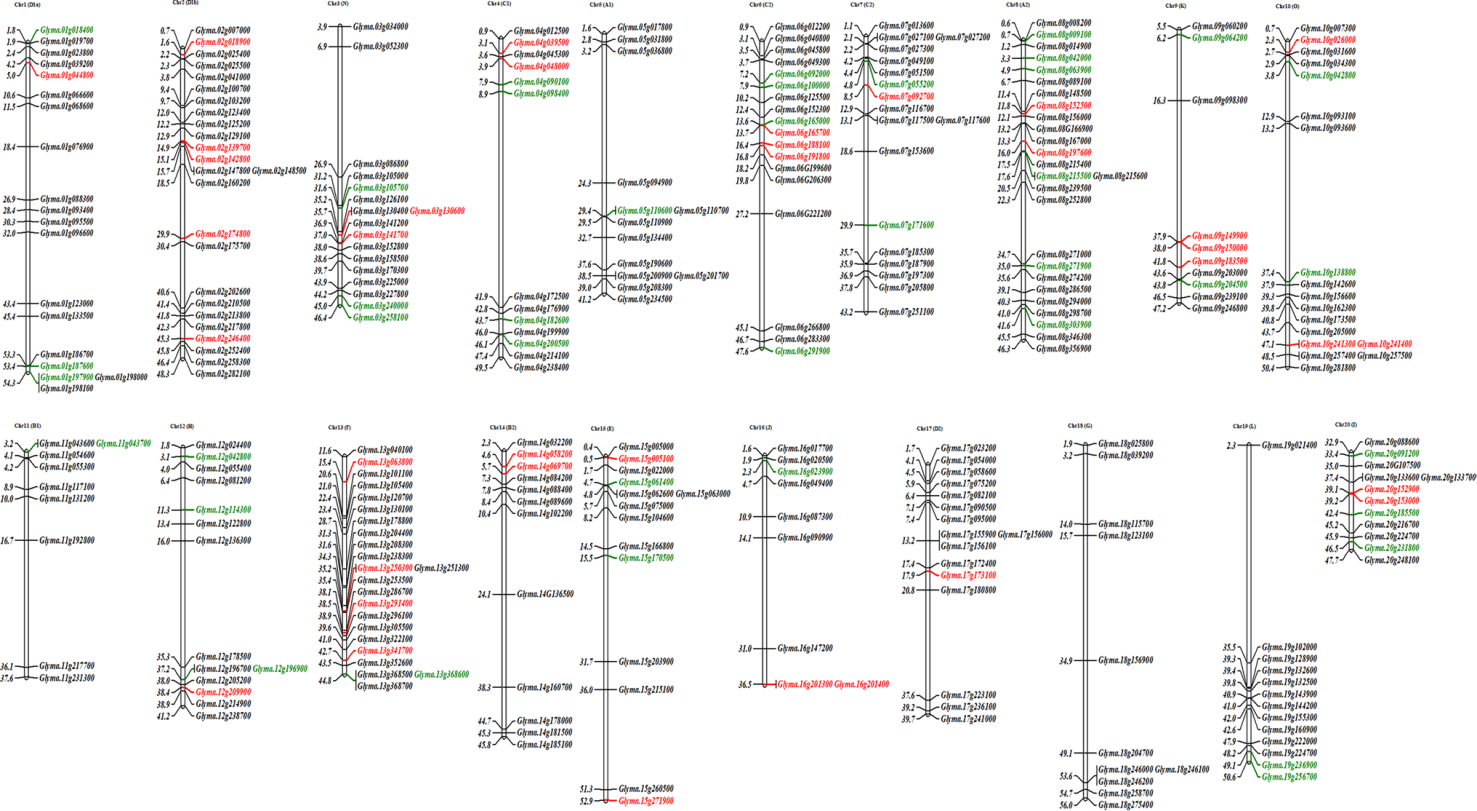
The figure displays the distribution of Glyma genes across soybean (*Glycine max*) chromosomes. The relative length of each chromosome is indicative of its size. Information regarding the location and chromosome details was sourced from Phytozome

In plants, the model organism *Arabidopsis* is commonly used to predict the function of a gene in a newly or partially sequenced organism that has a close phylogenetic relationship to *Arabidopsis*, such as soybean. Moreover, there are at vast number of WRKY in *Arabidopsis* and HLH gene family in soya bean that have been extensively studied and reported to be involved in many physiological and biochemical processes. After processing 56,044 sequences from Gmax_275_Wm82.a 2.v1.protein_primary Transcript Only 56,044 of input sequences are protein, 0 of input sequences are nucleotide, 4687 of proteins were identified as transcription factors or TRs, 2332 of proteins were identified as protein kinase. The TFs that were matched with reference genome were 69 involving 3903 genes % of total genes 56,044 and transcriptor regulators were 25 involving 784 genes and % of total genes 56,044. The location and presence of glyma gene family is given in Fig. 4.

**Fig. 5:**
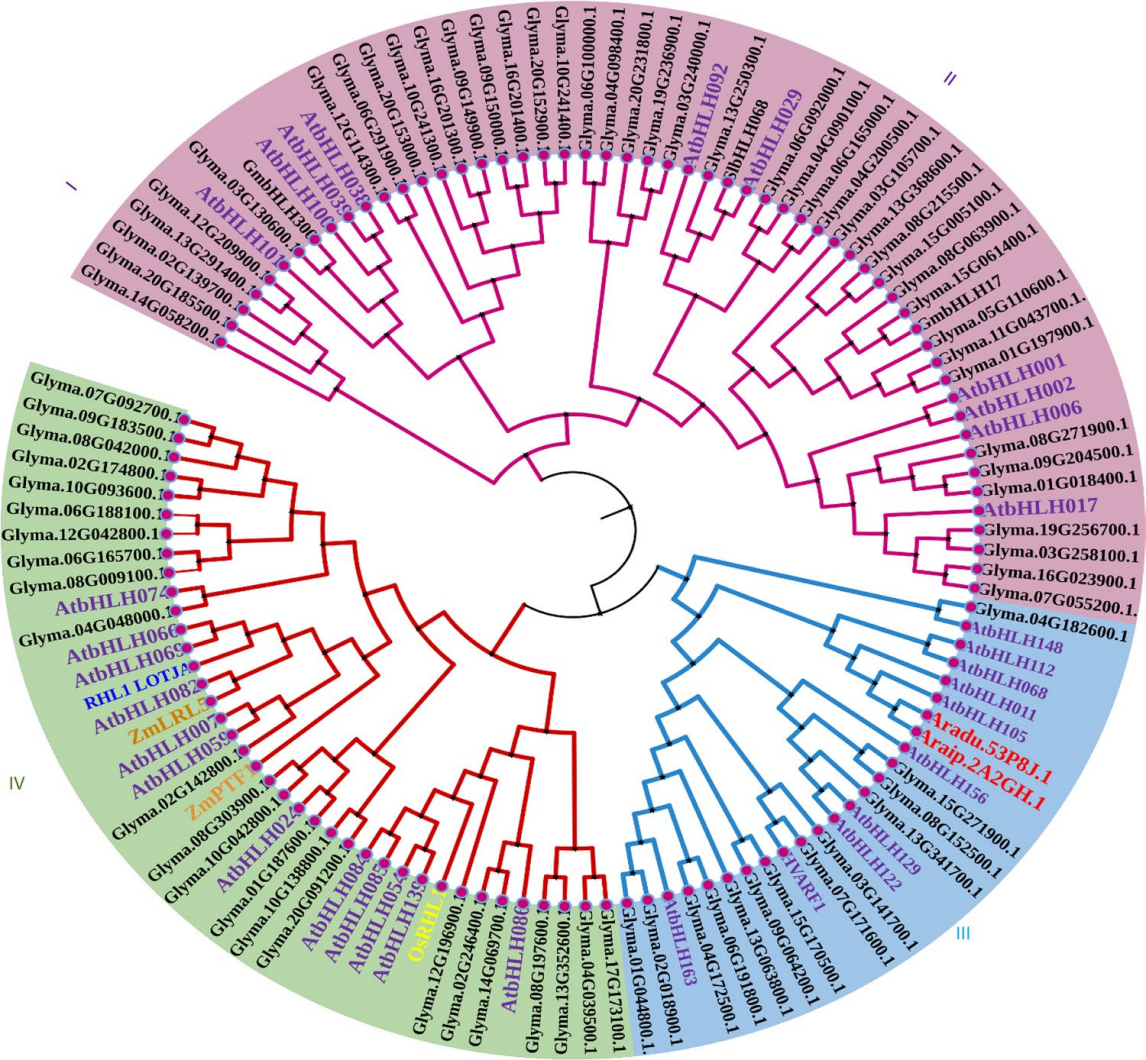
Phylogenetic tree depicting AtbHLH domains in soybean and *Arabidopsis*. Amino acid sequences were aligned using Clustal W, and the tree was constructed using the maximum likelihood method in MEGA 7.0. Gene expansion events in soybean and *Arabidopsis* are highlighted by coloring the respective subclades matching the leaf labels. Color codes denote distinct groups or subgroups within AtbHLH and Glyma domains, providing insights into evolutionary relationships and group-specific expansions

Furthermore, it is noteworthy that the Glyma domain’s structure and the resulting phylogenetic tree clearly delineated the distribution of Glyma and AtbHLH proteins across four main groups, each further branching into subgroups and clusters. Group I proteins exhibited a bifurcation into two distinct subgroups, while Group II was primarily characterized by division into two extensive subgroups, further branching into sub-subgroups and clusters. The observed phylogenetic classification was in concordance with the motif composition within each group or subgroup. Notably, distinctions between groups or subgroups were evident not only in the type of motifs present but also in their arrangement within individual AtbHLH proteins. Hence, the motif composition serves as an informative indicator of the phylogenetic relationships within the Glyma and HLH family, as illustrated in Fig. 5. This detailed analysis enhances our understanding of the evolutionary relationships and functional diversification among Glyma and AtbHLH proteins.

### DEG Seq analysis, GO and KEGG analysis

**Fig. 6:**
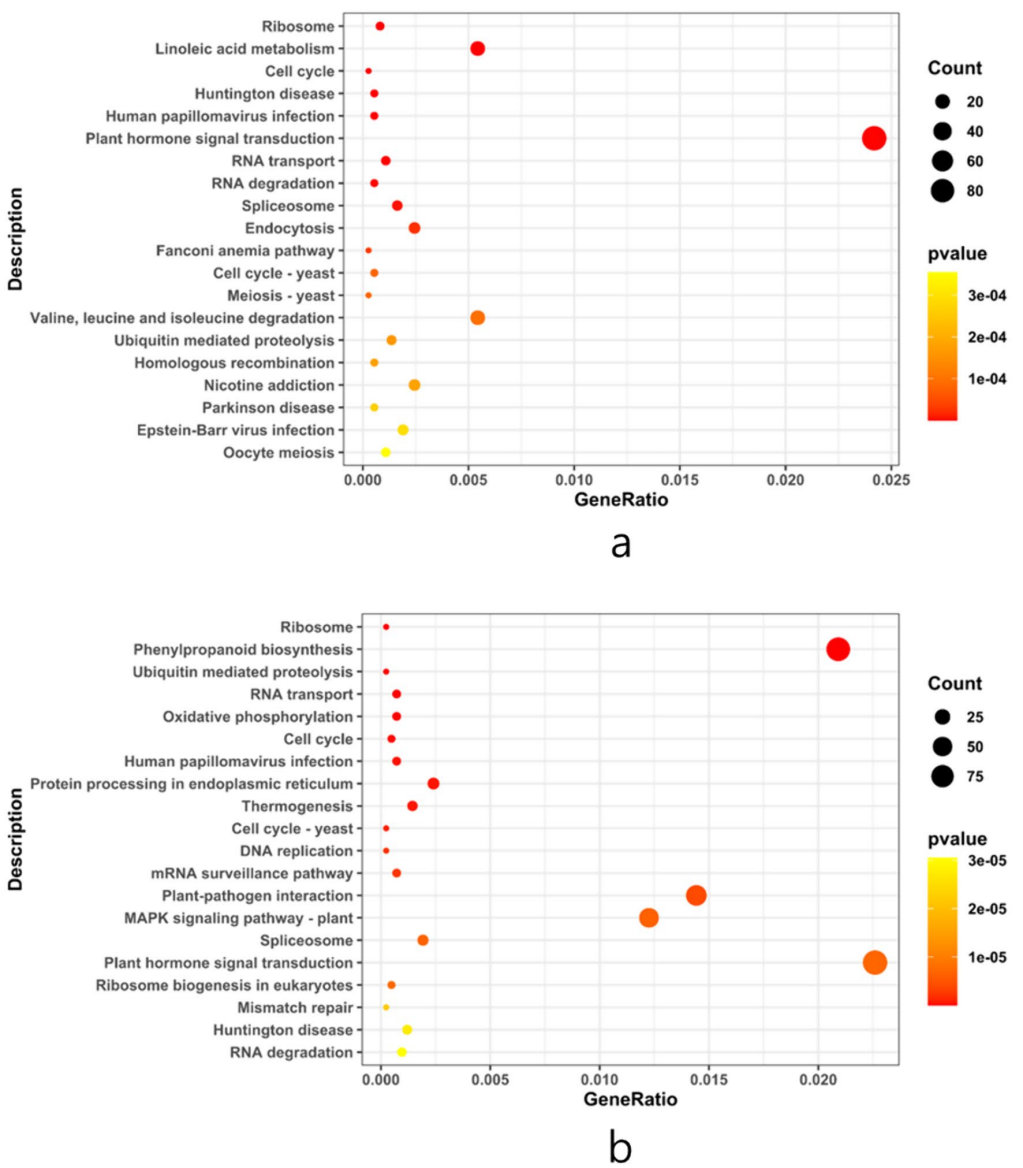
**a** Enrichment dot plot for upregulated pathways and **b** Enrichment dot plot for downregulated pathways. Illustration of the top 20 enriched pathways based on *P*-value in the enrichment_dotplot_up_top20 analysis. The color intensity and size of dots correspond to the significance levels, highlighting the pathways with the highest enrichment

The study conducted an analysis of Gene Ontology (GO) concentration in differentially expressed genes (DEGs) within soybean roots, focusing on three categories: Biological Process (BP), Cell Composition (CC), and Molecular Function (MF). The results, as illustrated in Fig. 6 and detailed in Supplementary File S2, revealed that the majority of DEGs in soybean roots were primarily associated with Molecular Function (MF), followed by Biological Process (BP) and Cell Composition (CC). The KEGG enrichment analysis was visually represented in a site map, showcasing the top 20 pathways with significant enrichment. If the number of enriched pathways was fewer than 20, all entries were presented. The evaluation of KEGG enrichment involved parameters such as Rich Factor (RF), False Discovery Rate (FDR), and the quantity of genes associated with each pathway.

**Fig. 7:**
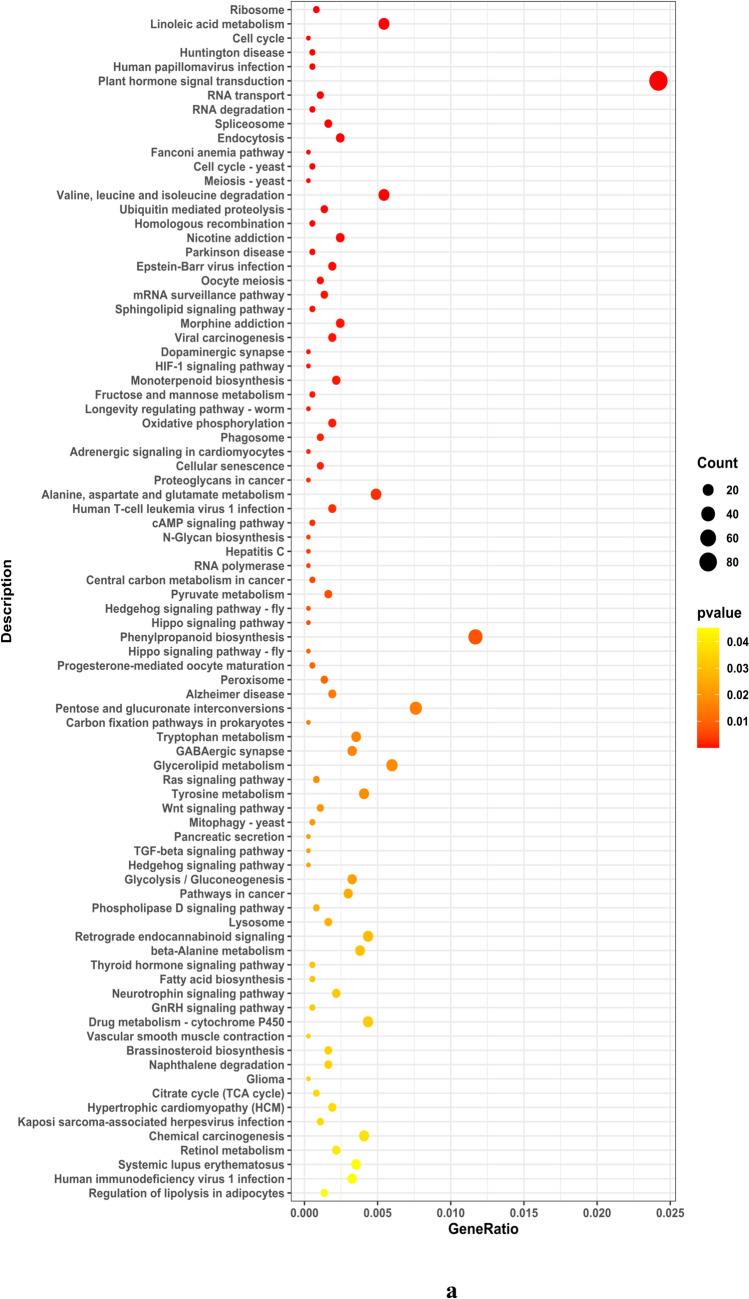

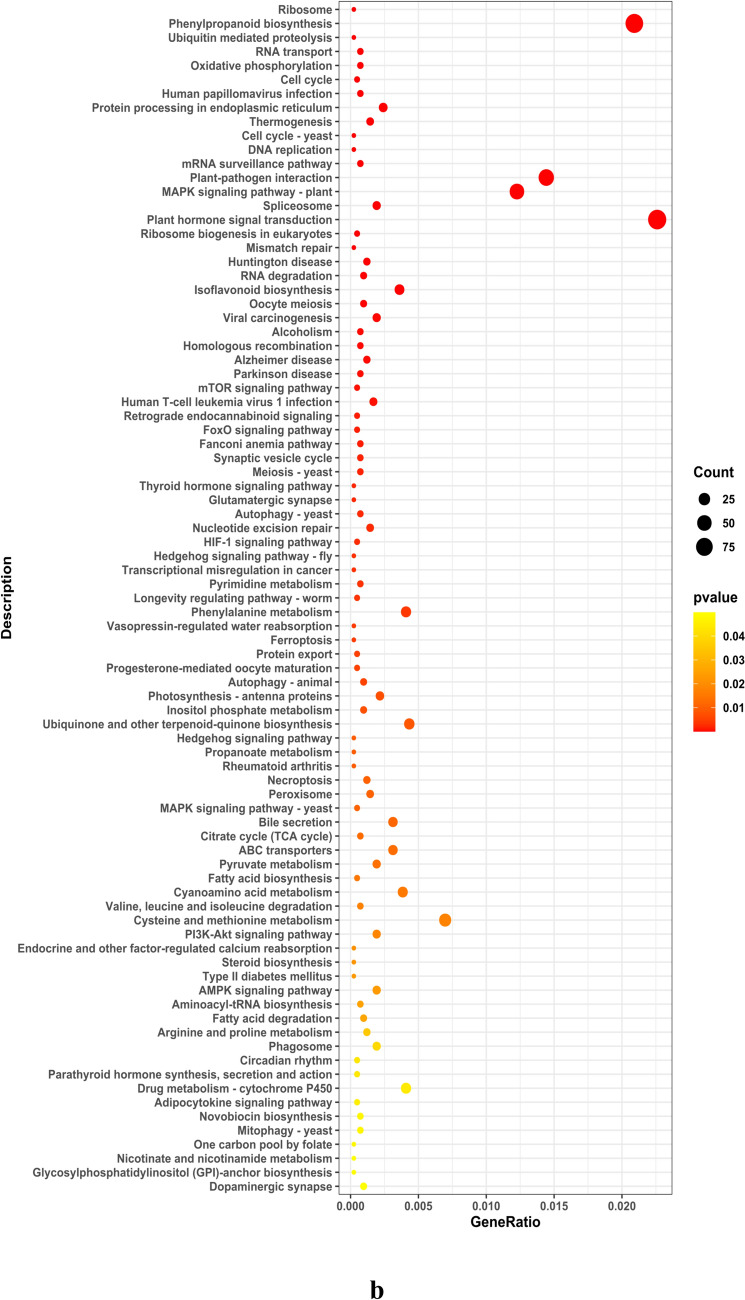
KEGG enrichment results for DEGs. **a** Enriched pathways for upregulated genes and **b** enriched pathways for downregulated genes. The analysis reveals the impact of gene expression changes on various biological processes and pathways. The color intensity represents the significance of enrichment

The RF serves as a metric representing the percentage of DEGs within a specific pathway entry, relative to the total number of genes in that pathway among all analyzed genes. A higher RF value indicates a greater concentration of DEGs within that particular pathway. Meanwhile, the FDR, ranging from 0 to 1, gauges the significance of enrichment, with values approaching zero signifying stronger enrichment. In this study, 188 upregulated genes were identified, showcasing diverse molecular functions including oxidoreductase activity, transcription regulator activity, DNA-binding TFs activity, transporter activity, transmembrane transporter activity, sequence-specific DNA binding, hydrolase activity acting on ester bonds, ion transmembrane transporter activity, and inorganic molecular entity transmembrane transporter activity. These molecular functions were found to be regulated by distinct sets of Glyma genes. The KEGG enrichment results for both upregulated (DEG UP) and downregulated (DEG DOWN) genes are visually presented in Fig. 7. This figure encapsulates the pathways exhibiting significant enrichment, shedding light on the molecular processes influenced by the experimental conditions and providing a comprehensive overview of the regulatory landscape within the studied system.

Twenty-six genes were found to be upregulated, overseeing CELLULAR_COMPONENT functions in soybean roots. Notably, genes associated with Casparian strip regulation, namely Glyma.02G200900.1.p, Glyma.13G315000.1.p, Glyma.10G075400.1.p, Glyma.19G083200.1.p, Glyma.10G075500.1.p, and Glyma.12G186500.1.p, exhibited high expression levels. Additionally, genes related to secondary cell wall control, including Glyma.02G200900.1.p, Glyma.13G315000.1.p, and Glyma.10G075400.1.p, were prominently expressed. Among the 316 genes regulating biological pathways, the key asparagine biosynthetic process was governed by Glyma.07G195300.1.p, Glyma.14G195000.1.p, Glyma.13G181000.1.p, and Glyma.12G150500.1.p. Furthermore, bundle sheath cell fate specification was directed by Glyma.09G270000.1.p and Glyma.18G220100.1.p, while cell junction assembly involved genes like Glyma.02G200900.1.p, Glyma.19G083200.1.p, Glyma.02G201100.1.p, Glyma.10G075500.1.p, and Glyma.12G186500.1.p. Citrate transport was controlled by Glyma.15G274600.1.p and Glyma.09G102800.1.p, while the regulation of unidimensional cell growth was governed by Glyma.02G143400.1.p and Glyma.19G031100.1.p. Additionally, root cap development was overseen by Glyma.15G266500.1.p and Glyma.08G161300.1.p.

Conversely, 336 genes were downregulated, influencing various biological processes, along with 27 genes impacting cellular components and 183 genes associated with molecular functions, as detailed in the Supplementary File S2. The downregulated genes were involved in functional groups such as catalytic activity, binding, metabolic processes, cellular processes, and biological regulation. These findings align with processes associated with root development, encompassing aspects like energy metabolism, nutrient accumulation, and cell proliferation. Genes with different expression were selected through comparative analysis of expression volume for reference gene regions. The analysis was performed using DESeq Normalized values based on the count values of the samples to be compared. Up/down regulation analysis was performed using Log2 Fold Change for genes calculated by satisfying the condition of ‘*P* value < 0.05’. Log2 fold change value is If it is above, it means that the expression amount is more than 2 times different, and it is judged that there is a significant difference between the comparison samples. Several genes within the AtbHLH family exhibited upregulation, overseeing key root functions (Table 2). Notably, AtbHLH105/ILR3, a member of the AtHLH family, was upregulated and plays a crucial role in regulating the network governing responses to wounding pathogens in plant roots. Additionally, it is involved in auxin-conjugate metabolism. Other genes, such as AtbHLH156/LHW, contribute to regulating the size of the vascular initial population in the root meristem, while AtbHLH024/SPT controls root growth by managing the size of the root meristem. AtbHLH74 was identified as a regulator of root growth in seedlings. Furthermore, bHLH066/LRL1 and bHLH069/LRL2 act redundantly to positively regulate the development of root hairs.

**Table 2:** Summary of differential expression of genes when comparing multiple cases and control groups

log2fold change ≥|1| & *p* value < 0.05			
A vs B	UP (B over expression)	DOWN (A over expression)	Total
(1_NO, 2_NO, 12_NO_mx) vs. (3, 4, 34_mx)	3,679	4,160	7,839

**Fig. 8:**
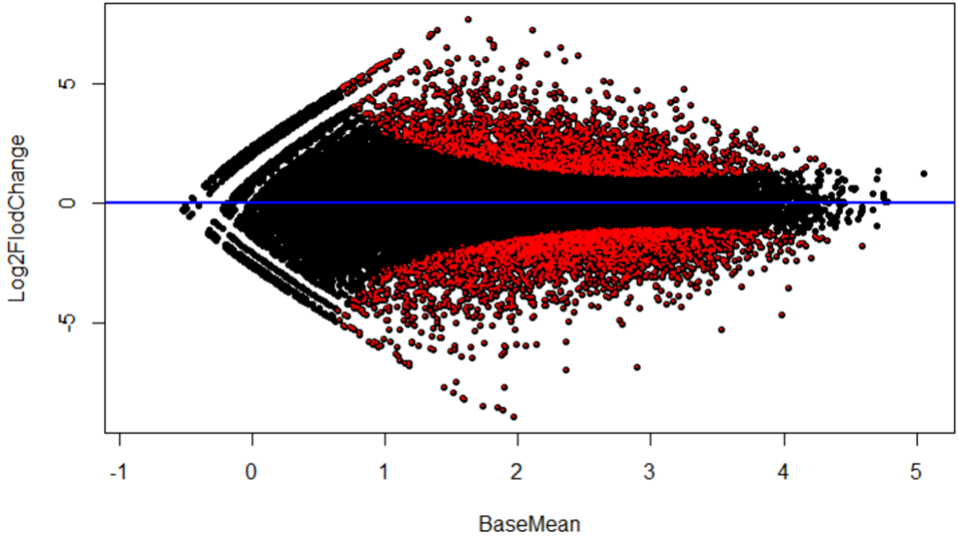
MA plot showing the expression difference between the two groups

The MA plot effectively shows the expression difference between the two groups. The Y-axis represents the Log2 Fold Change, the *X* axis represents the normalized mean expression value, and the *p*-value < *0* by statistical test. Genes that meet 05' are marked with a red dot (Fig. 8).

### TF sequence search results from the entire soy gene sequence with the iTAK

The iTAK analysis of protein sequences derived from 56,044 generations of soybeans revealed a comprehensive overview of TFs and TRs within the soy gene sequence. Out of the analyzed gene set, a total of 4687 TF and TR genes were identified (Table 3).

**Table 3:** List of transcription factors and transcriptional regulators

	TF-family	No. of genes	TR-family	No. of genes
1	Alfin-like	15	ARID	26
2	AP2/ERF-AP2	41	AUX/IAA	62
3	AP2/ERF-ERF	309	Coactivator p15	6
4	AP2/ERF-RAV	5	DDT	14
5	B3	70	GNAT	69
6	B3-ARF	56	HMG	21
7	BBR-BPC	10	IWS1	20
8	BES1	16	Jumonji	38
9	bHLH	313	LUG	12
10	BSD	0	MBF1	3
11	bZIP	144	MED6	1
12	C2C2-CO-like	23	MED7	2
13	C2C2-Dof	79	mTERF	57
14	C2C2-GATA	61	Others	133
15	C2C2-LSD	8	PHD	71
16	C2C2-YABBY	17	Pseudo ARR-B	12
17	C2H2	270	RB	3
18	C3H	115	Rcd1-like	6
19	CAMTA	15	SET	84
20	CPP	13	SNF2	64
21	CSD	8	SOH1	2
22	DBB	13	SWI/SNF-BAF60b	28
23	DBP	4	SWI/SNF-SWI3	6
24	E2F-DP	14	TAZ	9
25	EIL	12	TRAF	35
26	FAR1	77		
27	GARP-ARR-B	24		
28	GARP-G2-like	101		
29	GeBP	9		
30	GRAS	118		
31	GRF	22		
32	HB-BELL	34		
33	HB-HD-ZIP	96		
34	HB-KNOX	30		
35	HB-other	23		
36	HB-PHD	6		
37	HB-WOX	33		
38	HRT	1		
39	HSF	52		
40	LFY	2		
41	LIM	18		
42	LOB	91		
43	MADS-MIKC	88		
44	MADS-M-type	87		
45	MYB	330		
46	MYB-related	148		
47	NAC	179		
48	NF-X1	5		
49	NF-YA	21		
50	NF-YB	38		
51	NF-YC	23		
52	NOZZLE	2		
53	OFP	43		
54	PLATZ	30		
55	RWP-RK	28		
56	S1Fa-like	4		
57	SAP	2		
58	SBP	46		
59	SRS	21		
60	STAT	1		
61	TCP	56		
62	Tify	29		
63	Trihelix	64		
64	TUB	22		
65	ULT	17		
66	VOZ	6		
67	Whirly	7		
68	WRKY	185		
69	zf-HD	53		

Among these, 3903 genes were specifically classified as TFs, while 784 genes were categorized as TRs. This diverse set of TFs and TRs was further characterized into 68 distinct families, highlighting the complexity and diversity of regulatory elements present in soybeans. The identification of such a substantial number of TFs across various families underscores the intricate regulatory network governing gene expression in soybeans. These results contribute valuable insights into the transcriptional landscape of soybean genomes, providing a foundation for understanding the molecular mechanisms that underlie various biological processes and responses within this important crop species.

### Summary of expression values of transcription factors and transcription regulators

In the comprehensive analysis of TFs within the experimental context, distinct regulatory families exhibited differential expression patterns. Among the upregulated TFs, AP2/ERF-ERF 309, bHLH 313, bZIP 144, C2H2 270, WRKY185 and MYB 330. Among the TRs the number was AUX/IAA 62, GNAT 69, mTERF 57, SET84 and others 133. These TF genes exhibited varying expression values (FPKM) across the 1_NO, 2_NO, 12_NO_mx, 3, 4, and 34_mx samples. Similarly, among the downregulated DEGs, 481 TF genes were identified, and their expression values (FPKM) were documented across the same set of samples. The summary of expression values underscores the dynamic nature of TF regulation across experimental conditions. Notably, the diverse expression patterns within distinct TF families contribute to the intricate regulatory network governing gene expression in response to the experimental factors. These findings provide valuable insights into the transcriptional landscape, shedding light on the specific roles played by TFs in orchestrating molecular responses within the studied system.

### Validation of selected DEGs using qRT-PCR

**Fig. 9:**
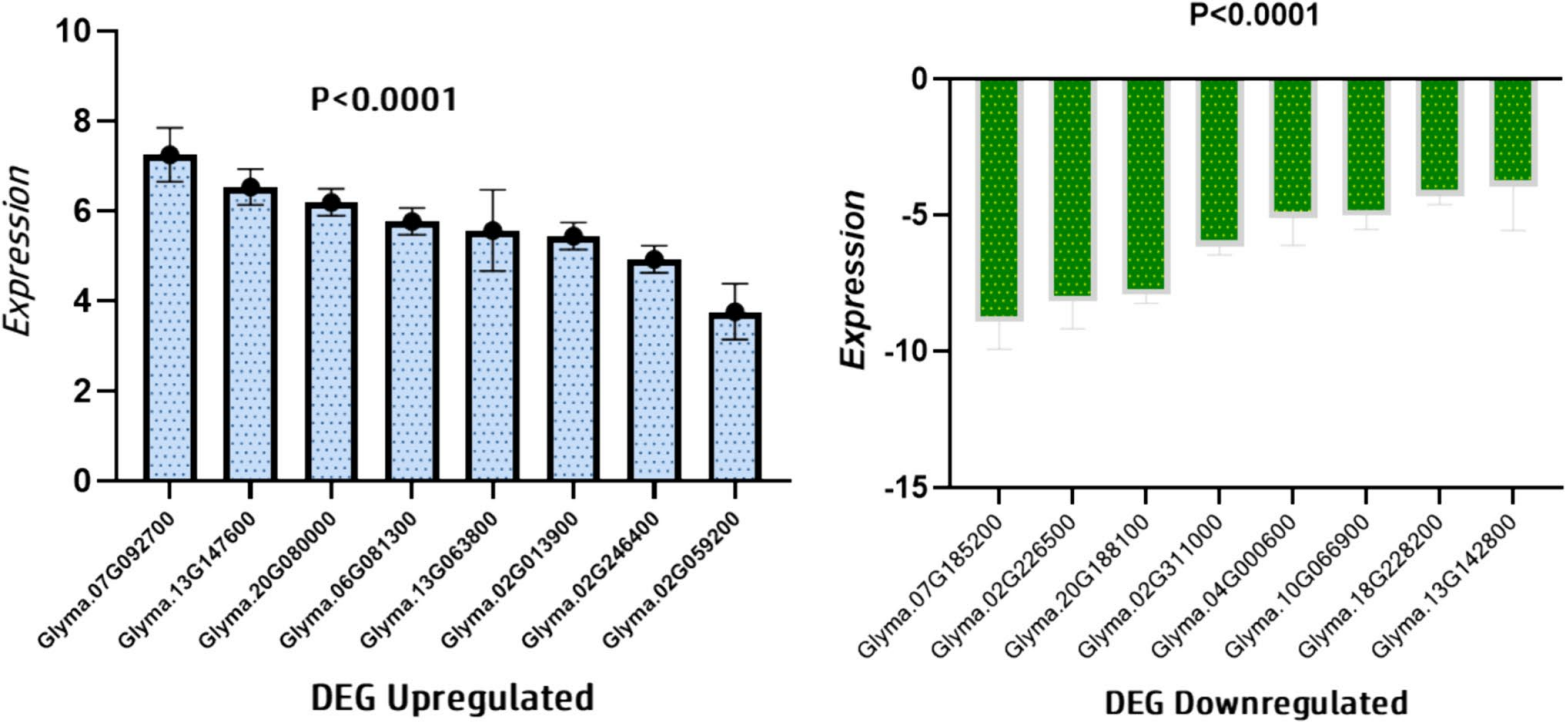
Validation of Differentially Expressed Genes (DEGs) Using qRT-PCR, Error bars, representing standard deviations, further emphasize the robustness of the qRT-PCR results, and statistical significance was determined using appropriate tests (*p < 0.05, **p < 0.01, ***p < *0.001*)

To validate the findings from our RNA-seq analysis, a subset of seven key DEGs associated with the basic Helix-Loop-Helix (bHLH) TFs, Transcription factor bHLH135, and hypothetical protein GLYMA_13G063800, as well as genes involved in amino acid synthesis, were selected for qRT-PCR analysis. The qRT-PCR results demonstrated a significant upregulation in the expression levels of Glyma.07G092700, Glyma.20G080000, and Glyma.06G081300. Conversely, Glyma.07G185200 exhibited a notable downregulation. Additionally, several other downregulated genes, namely Glyma.02G226500, Glyma.20G188100, Glyma.02G311000, and Glyma.04G000600, were identified (Fig. 9). These genes are associated with the expression of ethylene-responsive TFs ERF024-like, protein NLP2-like [*Glycine max*], vacuolar iron transporter 1-like, and auxin-induced protein X15-like [*Glycine max*], respectively. The figure illustrates the validation of identified DEGs through qRT-PCR. The DEGs were initially identified through transcriptome sequencing, and a subset of both upregulated and downregulated genes was selected for validation. The qRT-PCR results confirmed the expression changes observed in the transcriptome data, providing a reliable assessment of gene expression alterations. The upregulated genes displayed increased expression levels, while the downregulated genes exhibited decreased expression, aligning with the trends identified in the initial transcriptomic analysis. The validation through qRT-PCR enhances the confidence in the reliability and accuracy of the identified DEGs, reinforcing their significance in the context of the experimental conditions.

### Gene regulatory network

#### Co-expression modules

**Fig. 10:**
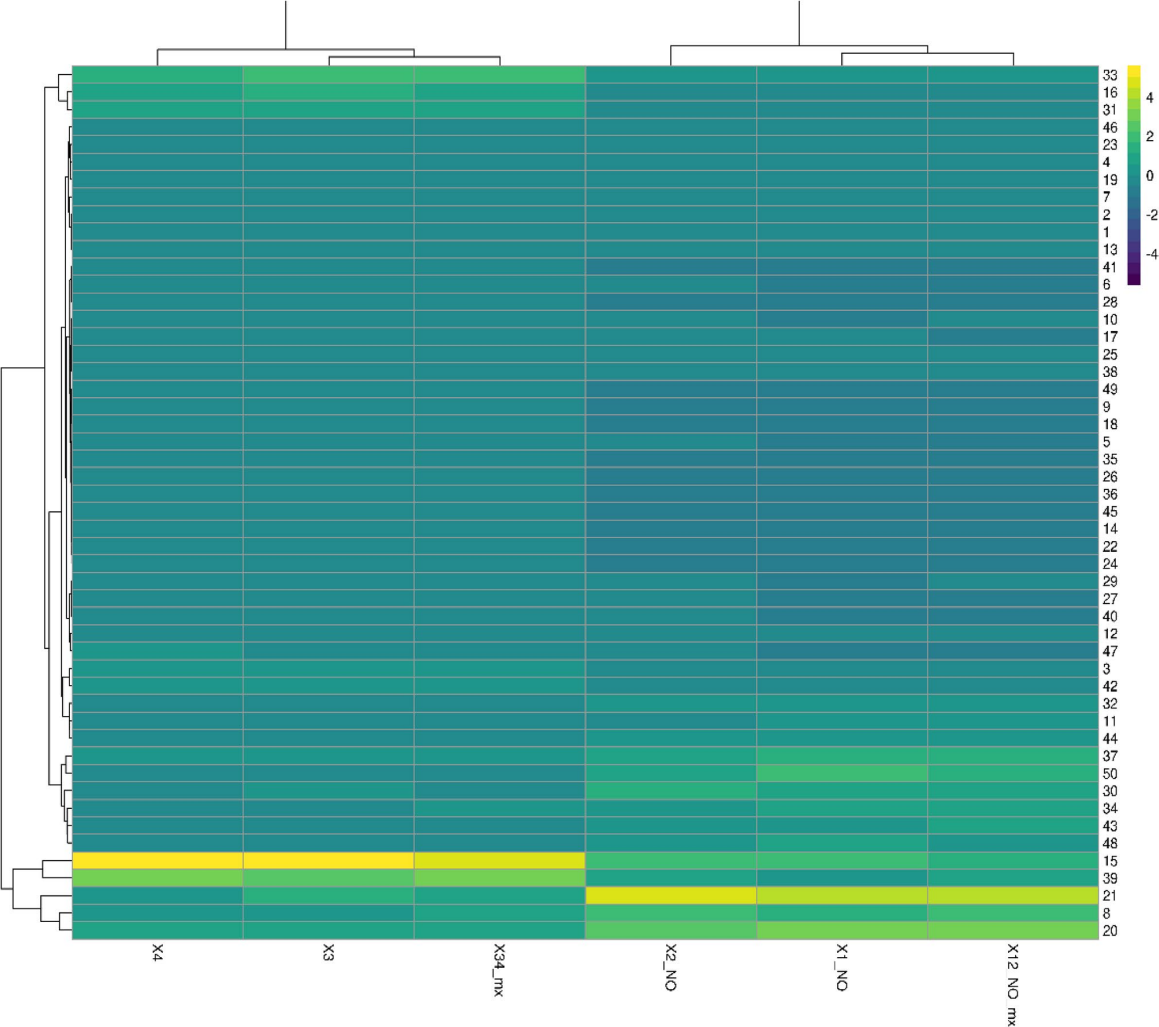
Heatmap of Clustering and gene function analysis. **A** Gene expression clusters and their co-expression pattern within different experimental condition. Color indicates normalized expression levels. Genes were clustered based on the K-means clustering algorithm. Hierarchical clustering was performed on the rows of the average expression levels for each cluster. Numbers on the right of the heatmap represent cluster id (CID, 1 to 50), with experimental conditions on the *x* axis

We identified 7839 genes that were differentially expressed in at least one of the comparisons and these genes were used for K-means clustering analysis to identify co-expressed gene modules. We found that the optimal number of clusters (modules) is 50 based on BIC (Supplementary File S1), K was set to be an integer number from 10 to 100 with an incremental step size of 5. For each *K* value, the minimum BIC was achieved with *K* = 50. The module 45 was found to be the largest cluster of 2253 genes with Glyma.01G001500 as a central gene and the smallest cluster is module 15 of 4 genes with Glyma.01G004000 as a central gene (Supplementary File S3). Average expression levels of all genes in each module for all experimental samples were used to generate a heat map (Fig. 10) as a co-expression pattern of modules within experimental condition.

### Regulatory network inference

**Fig. 11:**
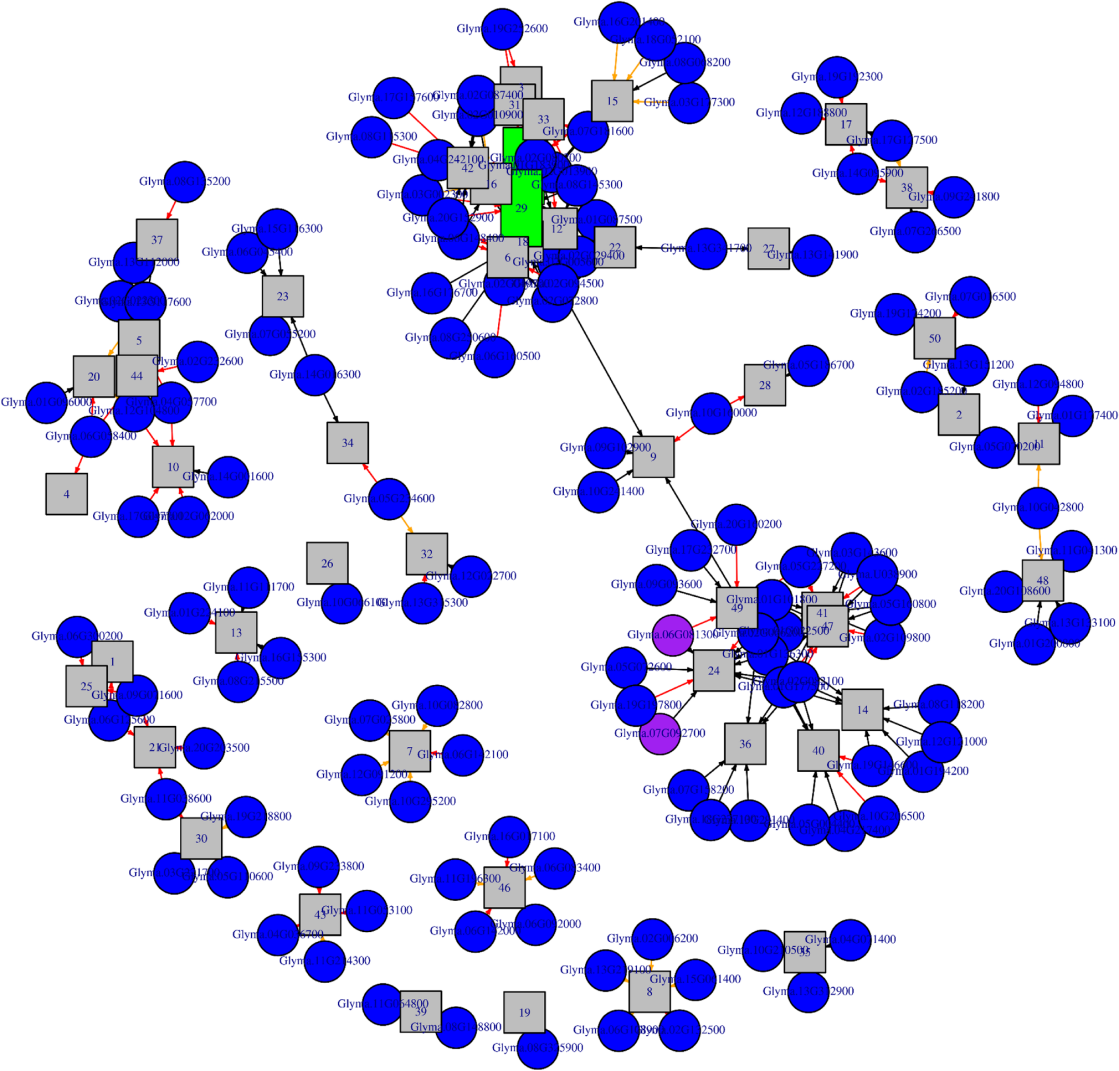
Gene regulatory network, where modules are in the form of squares and TFs as circles. Highly connected modules are highlighted with green colour and in the shape of a rectangle. Validated genes are highlighted with purple. Edges with significance score above 5 are highlighted with red colour and those with a significance score greater than 10 or lower than 10 are highlighted with orange

Five different network inference algorithms were used to infer putative regulatory interactions between regulators and their targets (Fig. 11). Only three interactions between 3 TFs and 3 modules were predicted by all five algorithms (Supplementary File S3 and S4). Some modules were predicted to be regulated by more than one TFs and vice versa. The identified interactions represent highly stringent predictions and are a very conservative estimation of all possible interactions since only 0.013% of all 23,716 possible interactions were found to be significant by all five computational methods. Two hundred seventy-Two interactions between 135 TFs and 49 modules were supported by four or more methods (Fig. 11, Supplementary File S3 and S4), representing a larger number for predicted regulatory interactions. The network figure includes directed edges, each directed edge connects a TFs with its targeted gene module. Such edge represents predicted regulatory interactions. The regulatory interactions (directed edges) are further classified based on the differential expression pattern of the regulatory TFs and modules (Supplementary File S1). We found module 29 as highly connected in our network also two of our upregulated differentially expressed genes validated by qtpcr Glyma.07G092700 of bHLH family is regulated in interaction with module 24 and Glyma.06G081300 is regulated in interaction with module 24 and 49. We then classified significant edges with score greater than 5 and highly significant with 10 as activators and -10 as suppressors.

## Discussion

Soybean, a globally significant grain and oil crop, offers a wealth of genomic and transcriptomic data that facilitate exploration of its functional diversity. A crucial aspect of this investigation is the application of Si as a fertilizer, recognized for its vital role in promoting economical and sustainable crop cultivation (Ning et al. 2014). The beneficial effects of Si, previously observed in rice, such as increased tiller numbers and enhanced resistance to abiotic stress, prompted the examination of its impact on soybean root nodulation and root development (Chung et al. 2020; Tripathi et al. 2021). The use of comparative transcriptomics allowed for the identification of alterations in gene expression patterns, with a specific focus on different DEGs associated with soybean root nodulation and development induced by Si application. The findings revealed 26 upregulated genes governing cellular component functions in soybean roots, indicating a significant impact of Si on the cellular processes underlying root development. Moreover, the study explored a comprehensive set of 316 genes involved in regulating biological and molecular pathways. The iTAK analysis of protein sequences derived from a vast number of soybean generations (56,044) provided insights into the TFs and TRs within the soy gene sequence. Remarkably, a total of 4687 TF and TR genes were identified, with 3903 genes specifically classified as TFs and 784 genes categorized as TRs (Supplementary File S5). This extensive set of TFs and TRs was further organized into 68 distinct families, highlighting the intricate and diverse regulatory elements present in soybeans. In a study conducted by Zhu et al. (2024), the GRAS TF family in kiwifruit (AcGRAS) was investigated for its response to salt stress. Among the 81 identified genes, 17 showed differential expression, with 13 being upregulated and four downregulated.

The introduction of Si fertilizer to the soil led to notable alterations in root phenotypic characteristics. Among the identified upregulated TFs, the AP2/ERF-RAV family featured 61 members, bHLH exhibited 38 members, bZIP showed 23 members, MYB demonstrated 38 members, and WRKY had 19 members. The study highlighted the significance of the auxin transporter pathway, along with the upregulation of processes such as the asparagine biosynthetic pathway, following Si application. Si’s involvement in isoflavonoid synthesis was underscored, with Si application to the soil resulting in increased nodule formation in soybean roots. The enrichment of the polypropanoid pathway among the upregulated genes in the KEGG analysis emphasized the pivotal role of Si in enhancing root nodulation in soybean cultivars. Additionally, the study identified upregulation in plant signal transduction pathways and metabolism pathways related to alanine, aspartate, and glutamate. Steiner et al. (2018), observed a two-fold increase in nodule number in soybeans with silicate fertilization. Nelwamondo and Dakora (1999) reported increased nodules and nodule dry matter in cowpea treated with metasilicic acid, attributing it to Si’s influence on nod gene expression, impacting nodule formation in leguminous plants. The synthesis of isoflavonoids, facilitated by Si, was also emphasized as a contributing factor to increased nodule formation in leguminous plants, as these plants release isoflavonoids to attract nitrogen-fixing bacteria (Van Bockhaven et al. 2013) Thus, underscores the multifaceted impact of Si on various molecular pathways and processes related to root nodulation in soybeans, providing valuable insights for agricultural practices. Tripathi et al. (2021) investigated the impact of Si application to both the soil and leaves of soybean, revealing a notable rise in nodule number and size. Additionally, Si-treated soybean plants exhibited significant alterations in root morphological traits compared to control plants.

Among the TFs that exhibited upregulation in our study, the AP2/ERF-RAV family comprised 61 members, bHLH showed 38 members, bZIP featured 23 members, MYB demonstrated 38 members, and WRKY included 19 members. The auxin transporter pathway emerged as a significant upregulated pathway, along with the asparagine biosynthetic process, following Si application. Wang et al. (2019) and Tripathi, et al. (2022) conducted a study investigating the expression levels of genes associated with root development and auxin transporter pathways in two distinct soybean cultivars. The Si-treated cultivar exhibited the highest expression levels for YUC3 and YUC5-1 genes compared to the control and Si-treated cultivar. YUCCA genes, belonging to the class B flavin-dependent monooxygenases, play a crucial role in the rate-limiting step of endogenous auxin synthesis, influencing plant growth regulation and stress response.

Our study also highlighted the role of specific bHLH genes in root development. For instance, AtbHLH156/LHW sub family plays a vital role in vascular cell establishment and the size of the vascular initial population in the root meristem. Additionally, AtbHLH024/SPT in S24 regulates root growth by controlling the size of the root meristem. Homologs of AtbHLH002/GL3, AtLRLs and AtRSLs are involved in root hair development. Furthermore, AtbHLH92 and AtbHLH129 regulate root elongation, while AtbHLH74 regulates seedling root growth. The current study conducted with soybean roots indicated high expression levels of AtbHLH orthologs of these functionally characterized bHLHs, suggesting their potential significance in root development. The *Arabidopsis* TF AtbHLH1, with prominent expression in roots, functions as a negative regulator of iron homeostasis (Gao et al. 2020). The bHLH family, known for its diverse physiological and developmental roles, particularly in metabolism and development, includes key players like AtbHLH045/MUTE in *Arabidopsis*, SlbHLH22 in tomato, and SPATULA (SPT) homologs in *Arabidopsis* and Prunus persica (Girin et al. 2011; Pillitteri et al. 2007; Tani et al. 2011; Waseem et al. 2019) Furthermore, certain bHLH members respond to various abiotic stresses. For instance, PHYTOCHROME-INTERACTING FACTOR 4 (PIF4) in *Arabidopsis* mediates plant stomatal response to high temperatures (Lau et al. 2018). StbHLH1 in potato regulates anthocyanin biosynthesis in response to high temperature (Liu et al. 2019), and MdbHLH3 in *Malus domestica* responds to low temperature (Xie et al. 2012). Moreover, bHLH TFs play a role in hormonal responses. *Arabidopsis* MYC2 is a well-known regulator in abscisic acid (ABA), jasmonic acid (JA), and light signaling pathways (Abe et al. 2003; Lorenzo et al. 2004; Yadav et al. 2005)Additionally, bHLHs such as JAMs and TT8 act as jasmonate-responsive TFs involved in secondary metabolism (Zhou and Memelink 2016). Among the upregulated genes, AtbHLH105/ILR3 was identified, responsible for regulating the network controlling wounding pathogen response in plant roots and participating in auxin-conjugate metabolism. The TF bHLH006/MYC2 has been identified in various plant species, including *Arabidopsis*, *Oryza sativa* (rice), *Solanum lycopersicum* (tomato), *Marchantia polymorpha*, *Catharanthus roseus*, *Salvia miltiorrhiza*, *Triticum aestivum* (wheat), *Artemisia annua*, and *Aquilaria sinensis*. In *Arabidopsis*, this TF is associated with the regulation of salt tolerance genes, specifically AtNHX6, in the roots. Additionally, bHLH006/MYC2 plays a role in promoting responsiveness to abscisic acid (ABA) and jasmonic acid (JA) (Du et al. 2018; Gupta et al. 2014; Krishnamurthy et al. 2019; Peñuelas et al. 2019; Zhang et al. 2011).

Other genes, such as AtbHLH156/LHW, were involved in regulating the size of the vascular initial population in the root meristem, while AtbHLH024/SPT played a role in regulating root growth by controlling the size of the root meristem. AtbHLH74 was associated with regulating root growth in seedlings, and bHLH066/LRL1 and bHLH069/LRL2 were also implicated in these processes. In a separate study by Ke et al. (2020), it was observed that the expression profiles of BnabHLHs changed significantly when roots were treated with five different hormones (IAA, auxin; GA3, gibberellin; 6-BA, cytokinin; ABA, abscisic acid; and ACC, ethylene). The induction of five candidate BnabHLHs was confirmed through qRT-PCR following the hormone treatments. A total of 246 BnabHLHs from nine subfamilies were predicted to have potential roles in root development based on joint analysis of their expression profiles and homolog functions. The TF bHLH002/GL3 is identified in various plant species, including *Arabidopsis, Arabisalpina, Gossypium hirsutum, Petunia hybrida*, and *Zea mays*. It plays a partially redundant role in regulating anthocyanin biosynthesis, trichome development, and root hair development (Bernhardt et al. 2003; Feller et al. 2006; Payne et al. 2000; B. Zhang et al. 2019; Zimmermann et al. 2004). In *Arabidopsis*, AtbHLH92 responds to NaCl, dehydration, mannitol, and cold treatments, and it is involved in regulating root elongation (Jiang et al. 2009). AtbHLH68 in *Arabidopsis* is associated with regulating ABA homeostasis and enhancing drought stress tolerance, while also influencing lateral root development (Le Hir et al. 2017; Bashir et al. 2021; Mansoor et al. 2022a, b). AtbHLH024/SPT controls root growth by overseeing the size of the root meristem (Makkena and Lamb 2013). Additionally, a group of bHLH proteins, including bHLH066/LRL1, bHLH069/LRL2, and bHLH082/LRL3, act redundantly to positively regulate the development of root hairs (Breuninger et al. 2016; Gajewska et al. 2018; Wang et al. 2019).

The control of unidimensional cell growth was governed by Glyma.02G143400.1.p and Glyma.19G031100.1.p. Additionally, Glyma.15G266500.1.p and Glyma.08G161300.1.p oversaw root cap development. In this investigation, 188 genes exhibiting upregulation were identified, showcasing diverse molecular functions, including oxidoreductase activity, transcription regulator activity, DNA-binding TF activity, transporter activity, transmembrane transporter activity, sequence-specific DNA binding, hydrolase activity acting on ester bonds, ion transmembrane transporter activity, and inorganic molecular entity transmembrane transporter activity. Distinct sets of Glyma genes were found to regulate these molecular functions. Glyma.06G102100 is homologous to the AtEXO gene, which responds to a brassinosteroid stimulus and is required for cell expansion in leaves (Schröder et al. 2009). Glyma.18G030200 is homologous to the COI1 gene, which is involved in jasmonate signaling and can inhibit growth and induce defense-related processes (Katsir et al. 2008). Glyma.19G016400 is a member of the ATP-binding cassette (ABC) transporter superfamily. This gene family has been associated with many functions of plant development and response, such as the transportation of auxin and secondary metabolites (Hwang et al. 2016). Glyma.03G160100, shared across 14 genotypes, is most homologous to AtCYP94B1, which is involved in apoplastic barrier formation in the roots and confers salt tolerance (Krishnamurthy et al. 2020).

For the qRT-PCR analysis, DEGs related to the basic Helix-Loop-Helix (bHLH) TFs, Transcription factor bHLH135, and the hypothetical protein GLYMA_13G063800, along with genes involved in amino acid synthesis, were specifically chosen. The results of qRT-PCR indicated a significant upregulation in the expression levels of Glyma.07G092700, Glyma.20G080000, and Glyma.06G081300. Conversely, Glyma.07G185200 showed a notable downregulation. Additionally, other downregulated genes, namely Glyma.02G226500, Glyma.20G188100, Glyma.02G311000, and Glyma.04G000600, were identified. These genes are associated with the expression of ethylene-responsive TF ERF024-like, protein NLP2-like [*Glycine max*], vacuolar iron transporter 1-like, and auxin-induced protein X15-like [*Glycine max*], respectively. The overall higher expression patterns in soybean cultivars suggested stronger expression in pathways related to root growth, auxin transporter, or cell development, potentially contributing to positive responses in root morphological traits. Previous studies by Manivannan and Ahn, (2017); Yin et al. (2016), which highlighted Si’s role in regulating critical genes involved in water transport, polyamine production, transcription regulation, defense, photosynthesis, and housekeeping under abiotic and biotic stresses.

Therefore, the current study reinforces the significance of Si in governing root development, nodulation, and physiological and oxidative activities in plants. Stress-response pathways, such as the phenylpropanoid pathway, as well as genes associated with antioxidants and phytohormones like abscisic acid, ethylene, and jasmonic acid, are indicated to be influenced by Si application in soybean.

Several enzymes participating in myo-inositol biosynthesis share regulatory patterns across plants, exemplified by the co-localization of inositol-polyphosphate 5-phosphatase (Glyma.20G170500) and inositol-phosphate phosphatase (Glyma.09G011100) in module 16. Within this module, genes exhibit upregulation in the mid-stages of root development, coupled with heightened expression during the early phases of both root and nodule development. Notably, a MYB-related TF in the same module bears resemblance to *Arabidopsis* RSM1, associated with auxin signalling in early morphogenesis. This coordinated regulatory response during mid-stages of root development suggests the potential significance of these enzymes and the associated TF in shaping early root and nodule development, implying intricate molecular processes in early morphogenesis. In the broader context, the conservative predictions, encompassing only 0.013% of conceivable interactions, highlight the intricacy of regulatory relationships. The analysis unveils complexity, with certain modules expected to be regulated by multiple TF, and vice versa, emphasizing the multifaceted nature of regulatory dynamics. The expanded set of 272 interactions, involving 135 TF and 49 modules, supported by four or more computational methods, introduces nuanced interplay, enhancing the understanding of the regulatory landscape. The meticulous computational approach elevates the reliability of identified interactions, providing valuable insights into the regulatory architecture governing the studied system. Overall, this information advances comprehension of myo-inositol biosynthesis regulatory networks and their role in plant development.

## Conclusion

In conclusion, the comprehensive investigation into the impact of Si application on soybean, a globally significant crop, has provided valuable insights into its multifaceted role in promoting root development, nodulation, and physiological activities in plants. The utilization the comparative transcriptomics, allowed for the identification of key genes and pathways influenced by Si. The study revealed significant alterations in gene expression patterns associated with cellular component functions, root development, and nodulation, highlighting the pivotal role of Si in shaping soybean’s functional diversity. The transcriptomic analysis uncovered a set of 316 genes regulating diverse biological and molecular pathways, with a focus on TFs TRs. The identification of 4687 TF and TR genes, further categorized into 68 distinct families, emphasized the intricate regulatory landscape in soybeans influenced by Si. The upregulation of specific TF families, such as AP2/ERF-RAV, bHLH, bZIP, MYB, and WRKY, along with the involvement of the auxin transporter pathway, underscored the molecular mechanisms contributing to enhanced root development and nodulation in response to Si application. Moreover, the study elucidated the enrichment of vital pathways, including the polypropanoid pathway, plant signal transduction, and metabolism pathways related to alanine, aspartate, and glutamate. These findings align with previous studies, corroborating Si’s positive influence on root morphology, nodule formation, and overall plant health. The collective evidence underscores the substantial role of Si in orchestrating complex molecular networks that contribute to soybean’s root development, nodulation, and overall physiological responses. These findings not only enhance our understanding of Si’s significance in sustainable agriculture but also offer practical insights for optimizing crop management strategies to enhance soybean yield and resilience in diverse environmental conditions.

### Supplementary Information

Below is the link to the electronic supplementary material.

Supplementary file1 (DOCX 1851 KB)

Supplementary file2 (XLSX 2917 KB)

Supplementary file3 (CSV 117 KB)

Supplementary file4 (CSV 33 KB)

Supplementary file5 (XLSX 1048 KB)

## Data Availability

The RNA-Seq data in this study have been deposited in National Center for Biotechnology Information (NCBI) under BioProject number PRJNA827666 and SRA accession numbers SRR18788739—SRR18788744.
