# Supplementary material for: Comparative transcriptomic analysis of the nodulation-competent zone and inference of transcription regulatory network in silicon applied Glycine max [L.]-Merr. Roots
Source: Plant Cell Rep. 2024 Jun 12;43(7):169. doi: 10.1007/s00299-024-03250-7 (PMC11169057; doi:10.1007/s00299-024-03250-7)
Supplement: Supplementary file 1 — Supplementary file1 (DOCX 1851 KB) [file 299_2024_3250_MOESM1_ESM.docx]

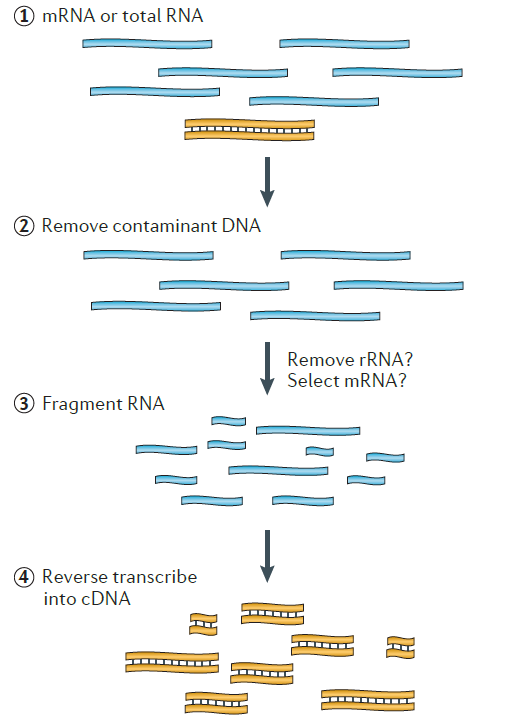

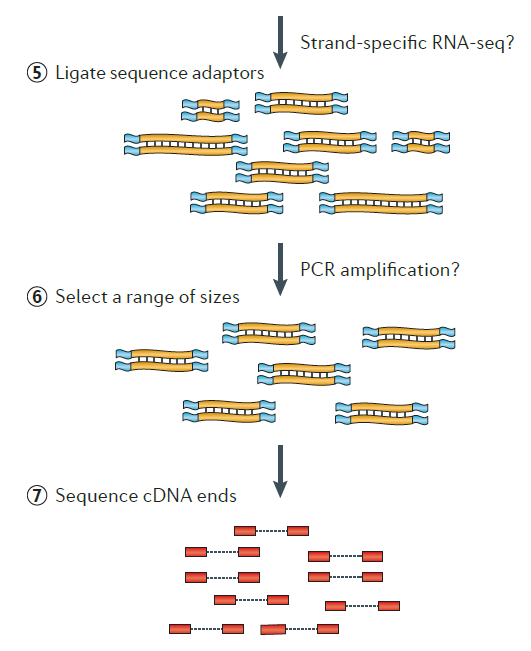


## Fig S1. RNA-Seq Data Generation Workflow

**Table: Reference Sequence Information**

| **IRGSP 정보** | **파일명** | **Total Sequence No.** | **Total Length (bp)** | **Min Length (bp)** | **Max Length (bp)** |
| --- | --- | --- | --- | --- | --- |
| Genome | Gmax_275_v2.0.fa | 1,190 | 978,495,272 | 1,002 | 58,018,742 |
| Transcript | Gmax_275_Wm82.a2.v1.transcript_primaryTranscriptOnly.fa | 56,044 | 92,028,847 | 87 | 16,968 |
| CDS | Gmax_275_Wm82.a2.v1.cds_primaryTranscriptOnly.fa | 56,044 | 65,462,595 | 87 | 16,308 |
| Protein | Gmax_275_Wm82.a2.v1.protein_primaryTranscriptOnly.fa | 56,044 | 21,820,865 | 29 | 5,436 |


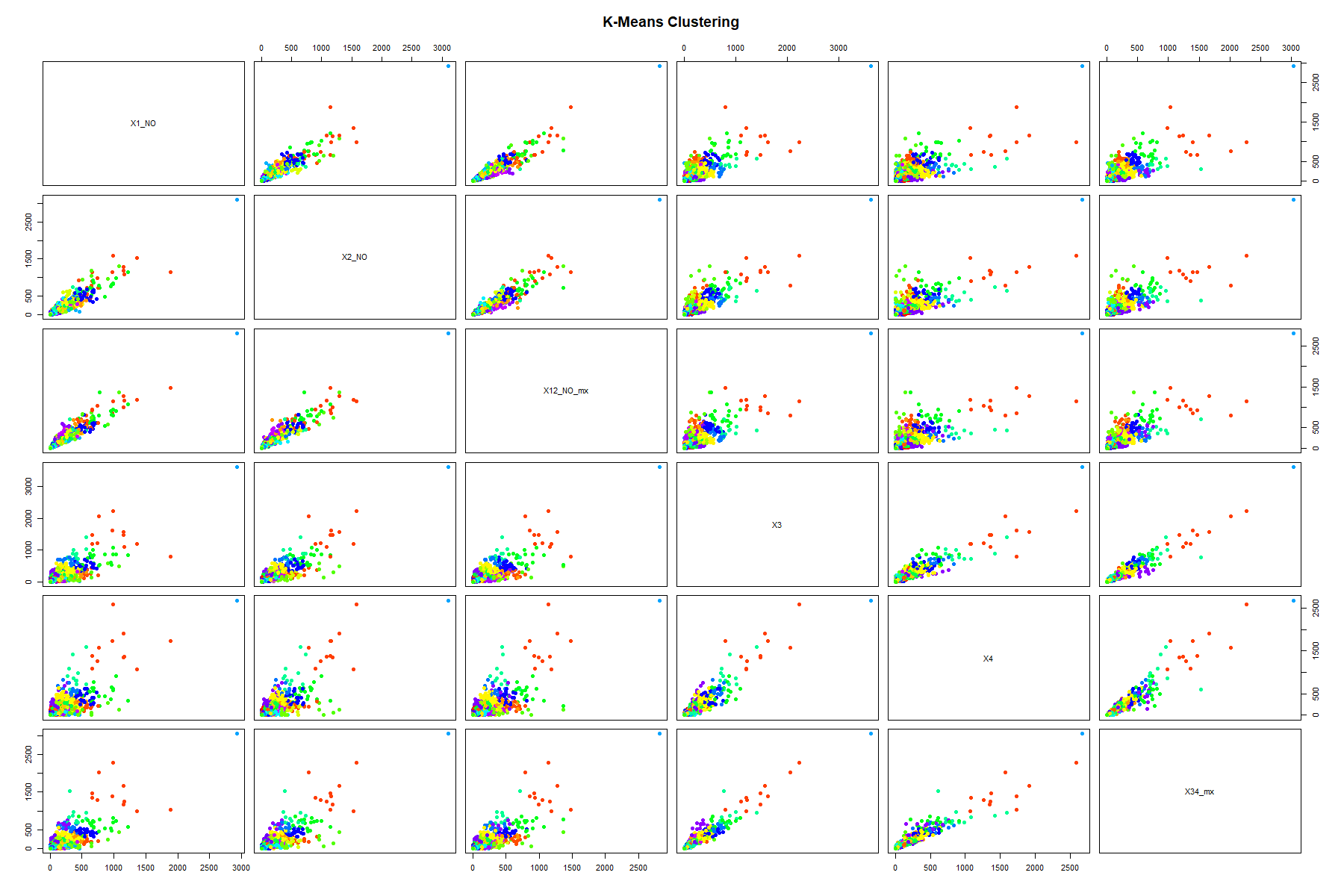


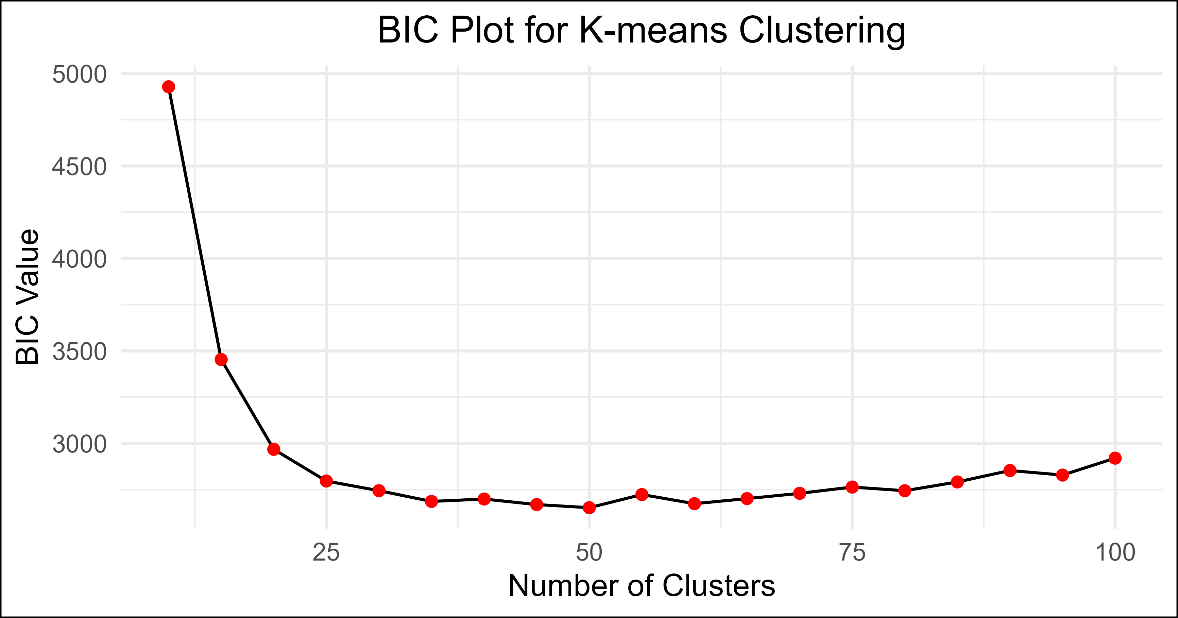


Figure: The data were subjected to k-means clustering analysis, and Bayesian Information Criterion (BIC) statistics were computed for different values of K. The plot illustrates the relationship between the number of clusters (K) and the corresponding BIC values. The minimum BIC, indicating the optimal number of clusters, was observed at K = 82.


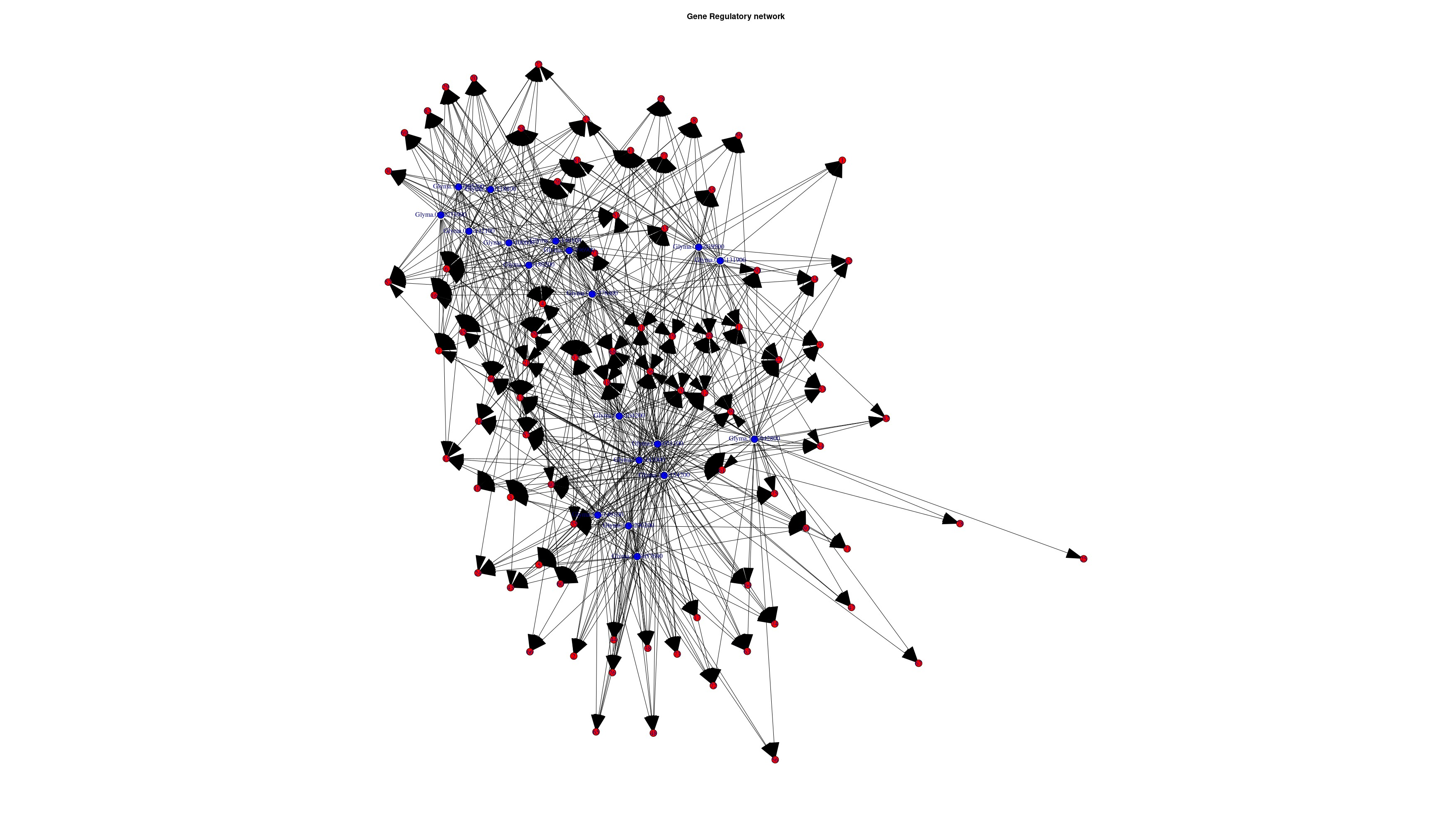


Fig: Gene Regulatory Network with edge
